# Does consistent motivation to stop smoking improve the explanation of recent quit attempts beyond current motivation? A cross-sectional study

**DOI:** 10.1016/j.addbeh.2018.01.037

**Published:** 2018-06

**Authors:** Olga Perski, Natalie Herd, Jamie Brown, Robert West

**Affiliations:** a Department of Clinical, Educational and Health Psychology, University College London, 1-19 Torrington Place, London WC1E 6BT, UK; b Cancer Research UK, Health Behaviour Research Centre, Department of Behavioural Science and Health, University College London, 1-19 Torrington Place, London WC1E 6BT, UK

**Keywords:** Smoking, Tobacco, Quit attempts, Current motivation to stop, Consistent motivation to stop, Cross-sectional

## Abstract

**Aims:**

In seeking to provide more accurate models of population quit attempt rates, this study assessed whether a single self-report measure of consistent motivation to stop smoking adds useful explanatory power over and above an established measure of current motivation to stop.

**Method:**

Data from 16,657 current smokers in England were collected between October 2012 and June 2017 using cross-sectional household surveys. Smokers were asked whether they had made a serious quit attempt in the past year and they answered two questions on motivation to stop (current motivation and consistent motivation to stop smoking). Having made at least one quit attempt in the past year was regressed in logistic models onto current motivation to stop and consistent motivation to stop individually and then together, with both models adjusting for sociodemographic variables and a measure of nicotine dependence.

**Results:**

The addition of consistent motivation to stop smoking added substantially to the fit of the model over and above the established measure of current motivation to quit (χ^2^(1, *N* = 16,657) = 901.7, *p* < 0.001) with an adjusted odds ratio of 4.1 (95% CI = 3.7–4.5, *p* < 0.001).

**Conclusion:**

Consistent motivation to stop smoking substantially improves the modelling of recent smoking cessation attempts over and above current motivation to stop. The consistency of smokers' motivation to quit may be a useful explanatory and target variable in future intervention studies.

## Highlights

• The addition of a single measure of ‘consistent motivation to stop’ may improve models of quit attempts.
• Consistent motivation to stop was strongly associated with having made a quit attempt in the past year.
• The consistency of the motivation to quit may be a useful target variable in intervention studies.
• The prospective association of consistent motivation to stop with quit attempts should be assessed in future research.

## 1 Introduction

It is important to continue to seek to develop improved models of smoking cessation attempts. Single self-report measures of current motivation to stop smoking are strongly associated with the likelihood of future quit attempts (Borland, Yong, Balmford, et al., 2010b; Smit, Fidler, & West, 2011). This paper addresses the issue of whether and how far adding a second type of measure relating to self-reports of consistent motivation to stop smoking in the past, may add to the explanatory power of models of quit attempts.

To develop improved interventions to increase the rate of quit attempts, a better understanding of what drives those attempts is needed. Motivation to stop is a key variable in this regard. Simple ratings of motivation to stop at a given point in time, as well as more complex ratings involving desire, intention and belief, have been found to be highly predictive of quit attempts in subsequent months (Borland, Yong, Balmford, et al., 2010b; Hummel, Brown, Willemsen, West, & Kotz, 2016; Kotz, Brown, & West, 2013; Vangeli, Stapleton, Smit, Borland, & West, 2011). These measures have only moderate stability over time (West, McEwen, Bolling, & Owen, 2001), which raises the question as to whether it is possible to construct a more accurate model of quit attempts by adding a further measure that asks smokers to reflect on and report the consistency of their motivation to stop. According to the PRIME theory of motivation, cessation attempts are expected to occur when momentary motivation to quit exceeds a given action threshold (West & Brown, 2013), which may be more likely to occur if it is experienced repeatedly over a period of time. To the extent that smokers are aware of, and able to report on, their motivation to quit in preceding months, this may add useful information to the measure of current motivation. To our knowledge, this idea has never been tested. A first step in doing so is to assess whether and how far a single self-report measure of consistent motivation to stop smoking improves the fit when regressing likelihood of having made at least one quit attempt in the past year onto an established measure of current motivation to stop.

## 2 Method

### 2.1 Study design and setting

The Strengthening the Reporting of Observational Studies in Epidemiology (STROBE) Initiative's checklist was used in the design and reporting of this study (Von Elm et al., 2007). This study is part of the “Smoking Toolkit Study”, an ongoing household survey designed to provide up-to-date information about smoking prevalence and cessation patterns in England. A series of monthly household surveys were conducted by trained interviewers. The sampling is a hybrid between random probability and simple quota, which has been shown to result in a sample that is representative of the general population of smokers in England (Fidler et al., 2011). Face-to-face computer-assisted interviews were held with one member of each household. Informed consent was obtained prior to each interview. Ethical approval for the Smoking Toolkit Study was provided by UCL's Research Ethics Committee.

### 2.2 Study population

Data included in the present study were collected from 16,657 respondents surveyed between October 2012 (the wave in which the measure of “consistent motivation to stop smoking” was added to the survey) and June 2017. As the variable “strength of urges to smoke” was assessed every other month between October 2012 and April 2013, data from respondents surveyed in November 2012, January 2013 and March 2013 were not included. Participants smoked cigarettes (manufactured or hand-rolled) or any other tobacco product (e.g. pipe, cigar) daily or occasionally at the time of the survey and were aged 16 or over. A post-hoc power calculation indicated that a total of 16,657 participants provided 90% power (two-tailed α = 0.05) to detect an odds ratio (OR) of 1.12 for the association between the dichotomous motivation scale (i.e. “consistent motivation to stop smoking”) and past quit attempts (Faul, Erdfelder, Lang, & Buchner, 2007).

### 2.3 Measures

The dependent variable was quit attempts made in the past 12 months, measured by asking: “How many serious attempts to stop smoking have you made in the past 12 months? By serious I mean you decided that you would try to make sure you never smoked again.” This item was coded 0 for smokers who responded that they had not made a quit attempt, and 1 for 1+ quit attempts. The independent variables were “consistent motivation to stop smoking” and “current motivation to stop smoking”. Consistent motivation to stop was measured by asking: “Have you consistently felt that you wanted to stop smoking in the past year?” The response options were: 1) No; 2) Yes. Current motivation to stop was measured by the Motivation to Stop Scale (Kotz et al., 2013), which asks: “Which of the following best describes you?” The response options were: 1) “I don't want to stop smoking”; 2) “I think I should stop smoking but don't really want to”; 3) “I want to stop smoking but haven't thought about when”; 4) “I REALLY want to stop smoking but I don't know when I will”; 5) “I want to stop smoking and hope to soon”; 6) “I REALLY want to stop smoking and intend to in the next 3 months”; 7) “I REALLY want to stop smoking and intend to in the next month”. This scale has been found to have at least as high a correlation with future quit attempts as other measures of current motivation to stop (Borland, Yong, O'Connor, Hyland, & Thompson, 2010a; Hummel et al., 2016; Hummel et al., 2017; Kotz et al., 2013; Kozlowski, Porter, Orleans, Pope, & Heatherton, 1994; Smit et al., 2011).

Respondents also provided data at baseline on age, sex and social grade (AB = managerial and professional occupations, C1 = intermediate occupations, C2 = small employers and own account workers, D = lower supervisory and technical occupations, and E = semi-routine and routine occupations, never workers, and long-term unemployed). Respondents reported their daily cigarette consumption and ratings of strength of urges to smoke as a measure of nicotine dependence with the question: “In general, how strong have the urges to smoke been?”. The response options ranged from 0 (None) to 5 (Extremely strong) (Fidler, Shahab, & West, 2011).

### 2.4 Analysis

To assess whether consistent motivation to stop smoking adds to the variance accounted for by current motivation to stop in explaining recent quit attempts (i.e., within the past 12 months), two multiple logistic regression models were fitted. The first model predicted recent quit attempts from current motivation to stop smoking. Consistent motivation to stop was added to the second model. Deviance statistics were calculated for both models by multiplying the log-likelihood by a factor of −2. The larger the deviance, the poorer the model fit. The difference between model deviances has a chi-square distribution with degrees of freedom equal to the difference in the number of parameters estimated. In both models, we adjusted for the following covariates: age, sex, social grade, cigarettes per day and strength of urges to smoke. Participants with missing data for any of the variables in the analyses were excluded. Data were analysed using SPSS version 21.0 (IBM Corp., 2012).

## 3 Results

A total of 17,460 smokers were surveyed between October 2012 and June 2017, of whom 16,657 (95.4%) provided complete data on all variables. Overall, 30.5% of smokers (n = 5080) had made an attempt to quit smoking in the past 12 months (see Table 1). Of the smokers who reported experiencing consistent motivation to stop, 54.1% (n = 4027) had made a quit attempt in the past year, compared with 11.4% (*n* = 1053) of smokers who reported that they had not experienced consistent motivation to stop.

**Table 1:** Baseline characteristics of participants (*N* = 16,657).

	% (N)
Sociodemographic characteristics	
Age	
16–24	19.8 (3298)
25–34	20.2 (3361)
35–44	16.5 (2752)
45–54	16.9 (2822)
55–64	14.0 (2327)
65+	12.6 (2097)
Female	47.0 (7836)
Social grade	
AB	11.0 (1838)
C1	26.3 (4373)
C2	23.3 (3883)
D	19.8 (3304)
E	19.6 (3259)

Smoking characteristics	
Current motivation to stop smoking	
“I don't want to stop smoking”	29.2 (4871)
“I think I should stop smoking but I don't really want to”	15.3 (2553)
“I want to stop smoking but haven't thought about when”	9.0 (1496)
“I REALLY want to stop smoking but I don't know when I will”	12.8 (2131)
“I want to stop smoking and hope to soon”	17.8 (2959)
“I REALLY want to stop smoking and intend to in the next 3 months”	7.8 (1302)
“I REALLY want to stop smoking and intend to in the next month”	8.1 (1345)
Consistent motivation to stop smoking	
No	55.3 (9207)
Yes	44.7 (7450)
Tried to quit in the past year	
No	69.5 (11,577)
Yes	30.5 (5080)
Strength of urges to smoke	
None	11.5 (1916)
Slight	15.8 (2633)
Moderate	45.3 (7540)
Strong	19.4 (3230)
Very strong	5.7 (948)
Extremely strong	2.3 (390)
Cigarettes smoked per day, mean *(SD)*	11.5 *(8.4)*

The addition of “consistent motivation to stop smoking” to a model including current motivation to stop smoking resulted in a substantial improvement of model fit (χ^2^(1, *N* = 16,657) = 901.7, *p* < 0.001). Smokers who reported that they had experienced consistent motivation to stop smoking were 4.1 times more likely to have made a quit attempt in the past year (95% CI = 3.7–4.5, *p* < 0.001) compared with smokers who reported that they had not experienced consistent motivation to stop, adjusting for current motivation to stop, age, sex, social grade, cigarettes per day and nicotine dependence as measured by strength of urges to smoke (see Table 2).

**Table 2:** Unadjusted and adjusted Odds Ratios (OR) of making a quit attempt in the past year for the different levels of current motivation to stop smoking and consistent motivation to stop smoking. *N* = 16,657.

	% Made a quit attempt in past year (n/N)	OR (95% CI)	OR^adj1^ (95% CI)	OR^adj2^ (95% CI)
Consistent motivation to stop smoking				
No	11.4 (1053/9207)	1.0	1.0	1.0
Yes	54.1 (4027/7450)	9.1 (8.4–9.9)⁎⁎⁎	4.0 (3.7–4.4)⁎⁎⁎	4.1 (3.7–4.5)⁎⁎⁎
Current motivation to stop smoking				
“I don't want to stop smoking”	6.8 (332/4871)	1.0	1.0	1.0
“I think I should stop smoking but don't really want to”	17.4 (445/2553)	2.9 (2.5–3.4)⁎⁎⁎	2.0 (1.7–2.4)⁎⁎⁎	2.0 (1.7–2.3)⁎⁎⁎
“I want to stop smoking but haven't thought about when”	20.7 (310/1496)	3.6 (3.0–4.2)⁎⁎⁎	2.1 (1.7–2.5)⁎⁎⁎	2.0 (1.7–2.4)⁎⁎⁎
“I REALLY want to stop smoking but I don't know when I will”	40.0 (852/2131)	9.1 (7.9–10.5)⁎⁎⁎	3.8 (3.2–4.4)⁎⁎⁎	3.7 (3.2–4.3)⁎⁎⁎
“I want to stop smoking and hope to soon”	49.7 (1472/2959)	13.5 (11.9–15.5)⁎⁎⁎	5.3 (4.5–6.1)⁎⁎⁎	5.0 (4.3–5.8)⁎⁎⁎
“I REALLY want to stop smoking and intend to in <3 months”	57.9 (754/1302)	18.8 (16.1–22.0)⁎⁎⁎	6.6 (5.6–7.9)⁎⁎⁎	6.3 (5.3–7.5)⁎⁎⁎
“I REALLY want to stop smoking and intend to in <1 month”	68.0 (915/1345)	29.1 (24.8–34.1)⁎⁎⁎	10.3 (8.6–12.2)⁎⁎⁎	9.8 (8.2–11.7)⁎⁎⁎
Age				
16–24	33.4 (1100/3298)	1.0	−	1.0
25–34	34.8 (1169/3361)	1.1 (0.96–1.2)	−	0.9 (0.8–1.0)
35–44	33.8 (930/2752)	1.0 (0.9–1.1)	−	0.8 (0.7–0.9)⁎⁎⁎
45–54	28.9 (816/2822)	0.8 (0.7–0.9)⁎⁎⁎	−	0.7 (0.6–0.74)⁎⁎⁎
55–64	26.2 (610/2327)	0.7 (0.6–0.8)⁎⁎⁎	−	0.6 (0.5–0.7)⁎⁎⁎
65+	21.7 (455/2097)	0.6 (0.5–0.6)⁎⁎⁎	−	0.6 (0.5–0.7)⁎⁎⁎
Sex				
Male	29.2 (2577/8821)	1.0	−	1.0
Female	31.9 (2503/7836)	1.1 (1.1–1.2)⁎⁎⁎	−	1.1 (0.97–1.13)
Social grade				
AB	33.6 (617/1838)	1.0	−	1.0
C1	31.6 (1381/4373)	0.9 (0.8–1.0)	−	0.9 (0.8–1.1)
C2	29.6 (1151/3883)	0.8 (0.7–0.9)⁎⁎	−	1.0 (0.9–1.1)
D	28.6 (945/3304)	0.8 (0.7–0.9)⁎⁎⁎	−	0.9 (0.8–1.0)
E	30.3 (986/3259)	0.9 (0.8–0.97)⁎	−	0.9 (0.8–1.1)
Cigarettes per day	−	0.985 (0.98–0.99)⁎⁎⁎	−	0.99 (0.985–0.995)⁎⁎⁎
Strength of urges to smoke				
None	21.2 (406/1916)	1.0	−	1.0
Slight	28.5 (751/2633)	1.5 (1.3–1.7)⁎⁎⁎	−	1.1 (0.96–1.3)
Moderate	31.8 (2400/7540)	1.7 (1.5–2.0)⁎⁎⁎	−	1.4 (1.2–1.7)⁎⁎⁎
Strong	33.0 (1065/3230)	1.8 (1.6–2.1)⁎⁎⁎	−	1.6 (1.3–1.8)⁎⁎⁎
Very strong	34.5 (327/948)	2.0 (1.6–2.3)⁎⁎⁎	−	1.8 (1.4–2.2)⁎⁎⁎
Extremely strong	33.6 (131/390)	1.9 (1.5–2.4)⁎⁎⁎	−	2.6 (1.9–3.5)⁎⁎⁎

^adj1^ OR adjusted for current motivation to stop smoking.

^adj2^ OR adjusted for current motivation to stop smoking, age, sex, social grade, cigarettes per day, strength of urges to smoke.

⁎ *p* < 0.05.

⁎⁎ *p* < 0.01.

⁎⁎⁎ *p* < 0.001.

## 4 Discussion

This study found that a single self-report measure of consistent motivation to stop smoking showed a strong association with the likelihood of having made at least one quit attempt in the past 12 months and added substantially over and above an established measure of current motivation to stop smoking. These findings suggest that smokers have enough recollection of the consistency of their motivation to quit over time for this to be used as an additional measure of motivation to stop smoking that could be useful in understanding quit attempts. These findings also lend support to phase-based models of quitting, which suggest that predictors of quit attempts may differ from predictors of cessation success (Hughes et al., 2014; Vangeli et al., 2011).

The present findings have methodological and theoretical implications. Firstly, since the consistency of the motivation to stop smoking adds to an established motivation to stop measure, it may be a useful explanatory and target variable in future intervention studies. For example, as environmental cues that remind smokers of their motivation to quit have been found to prompt quit attempts in observational studies (Hughes, Naud, Fingar, Callas, & Solomon, 2015), such environmental cues could be included in future intervention studies with a view to targeting consistent motivation to stop. Secondly, according to the PRIME theory of motivation, quit attempts are hypothesised to arise when momentary motivation to quit exceeds a given action threshold (West & Brown, 2013). The finding that consistency is an important dimension of motivation that could improve the explanatory and perhaps also the predictive power of models of quitting lends support to this hypothesis, suggesting that motivation to stop smoking may be more likely to exceed the threshold for action over a given time period if it is experienced repeatedly during that time.

The present study benefited from the inclusion of a large, nationally representative sample of smokers in addition to the use of a validated measure of current motivation to stop smoking. However, as the present study was limited by a cross-sectional design, it is not possible to determine the prospective association between consistent motivation to stop smoking and quit attempts made at a later point in time. Another limitation was the reliance on self-reported quitting data. As previous research indicates that retrospective recall of quit attempts may be inaccurate (Berg et al., 2010; Hughes et al., 2014), we cannot also rule out the possibility that retrospective recall of consistent motivation to stop is also inaccurate. Moreover, it is possible that smokers' memory of trying to quit but failing to do so, and their memory of having experienced consistent motivation to quit, may be influenced by one another. For example, smokers who recall having made several quit attempts over the past year may infer that they must have experienced consistent motivation to quit. Although the use of a dichotomous measure of consistent motivation to stop aided interpretation of the findings, a continuous measure would have been more informative and increased statistical power (Altman & Royston, 2006). Finally, we cannot assume that findings from the present study are generalisable to populations other than the general population of English smokers.

Future research should assess whether consistent motivation to stop smoking improves the prediction of future quit attempts using a prospective design. It should also be examined whether the consistency of smokers' motivation to quit can be specified further. For example, smokers who recall having experienced motivation to quit on a daily basis in the past year may be more prone to action than those who recall having experienced motivation to quit on a weekly or monthly basis. Future research could also include a wider range of smoking and sociodemographic characteristics to assess the extent to which consistent motivation uniquely improves the modelling of quit attempts, over and above other characteristics to which it may be related (e.g. length of smoking).

In conclusion, a single self-report measure of consistent motivation to stop smoking substantially improves the modelling of recent smoking cessation attempts over and above current motivation to stop. The consistency of smokers' motivation to quit may be a useful explanatory and target variable in future intervention studies.

## Role of funding sources

Cancer Research UK is the main contributor (C1417/A22962), but the UK Department of Health, Pfizer, GlaxoSmithKline and Johnson and Johnson have also all contributed funding to the data collection for the Smoking Toolkit Study. OP is funded by Bupa under its partnership with University College London. NH is funded by the NIHR HPRU in Evaluation of Interventions. RW and JB receive support from CRUK (C1417/A22962). The funders had no final role in the study design; in the collection, analysis and interpretation of data; in the writing of the report; or in the decision to submit the paper for publication. All researchers listed as authors are independent from the funders and all final decisions about the research were taken by the investigators and were unrestricted.

## Contributors

All authors contributed to the design of the study. OP conducted the statistical analysis and wrote the first draft of the manuscript. All authors have contributed to and have approved the final manuscript.

## Conflict of interest

OP and NH report no competing interests to declare. RW undertakes research and consultancy for and receives travel funds and hospitality from manufacturers of smoking cessation medications (Pfizer, GlaxoSmithKline and Johnson and Johnson). JB has received unrestricted research funding from Pfizer.
